# Contraceptive Use Among Women With End-Stage Kidney Disease on Dialysis in the United States

**DOI:** 10.1016/j.xkme.2020.08.010

**Published:** 2020-10-27

**Authors:** Silvi Shah, Annette L. Christianson, Charuhas V. Thakar, Samantha Kramer, Karthikeyan Meganathan, Anthony C. Leonard

**Affiliations:** 1 Division of Nephrology, Kidney C.A.R.E. (Clinical Advancement, Research and Education) Program, University of Cincinnati, Cincinnati, OH; 2 Division of Biostatistics and Bioinformatics, University of Cincinnati, Cincinnati, OH; 3 Cincinnati Veteran Affairs Medical Center, Cincinnati, OH; 4 Department of Family and Community Medicine, University of Cincinnati, Cincinnati, OH

**Keywords:** Rates, contraception, dialysis, race/ethnicity, end-stage kidney disease

## Abstract

**Rationale & Objective:**

Although end-stage kidney disease (ESKD) adversely affects fertility, pregnancies can occur among women receiving dialysis. ESKD increases the risk for adverse pregnancy outcomes and little is known about contraceptive use in women undergoing dialysis.

**Study Design:**

Retrospective cohort study.

**Setting & Participants:**

Using the US Renal Data System covering January 1, 2005, through December 31, 2014, we evaluated for each calendar year women who for the entire year were aged 15 to 44 years, receiving dialysis, and with Medicare as the primary payer.

**Predictors:**

Age, race/ethnicity, and calendar year of prevalent ESKD.

**Outcome:**

Contraceptive use.

**Analytic Approach:**

We determined rates of contraceptive use and used multivariable logistic regression to identify factors associated with contraceptive use.

**Results:**

The study cohort included 35,732 women and represented 115,713 person-years. The rate of contraceptive use was 5.30% of person-years (95% CI, 5.17%-5.42%). Overall, contraceptive use increased from 2005 to 2014 (4.21%; 95% CI, 3.84%-4.59% vs 6.54%, 95% CI, 6.10%-6.99%). Compared with women aged 25 to 29 years, contraceptive use was higher in women aged 15 to 24 years (OR, 1.30; 95% CI, 1.18-1.43) and lower in women aged 30 to 34 years (OR, 0.74; 95% CI, 0.68-0.81), 35 to 39 years (OR, 0.46; 95% CI, 0.42-0.50), and 40 to 44 years (OR, 0.30; 95% CI, 0.27-0.34). Compared with White women, contraceptive use was higher in Black (OR, 1.12; 95% CI, 1.02-1.24) and Native American women (OR, 1.60; 95% CI, 1.25-2.05). Women with ESKD due to glomerulonephritis had a higher likelihood of contraceptive use than women with ESKD due to diabetes (OR, 1.22; 95% CI, 1.06-1.42). Women receiving peritoneal dialysis had a lower likelihood of contraceptive use than women receiving hemodialysis (OR, 0.85; 95% CI, 0.78-0.93). Compared with women without predialysis nephrology care, contraceptive use was higher in women who received predialysis nephrology care for 12 or fewer months (OR, 1.22; 95% CI, 1.09-1.37) and more than 12 months (OR, 1.33; 95% CI, 1.20-1.47).

**Limitations:**

Retrospective design and use of administrative data.

**Conclusions:**

Among women with ESKD undergoing dialysis, contraceptive use remains low at 5.30%. Younger age, Native American and Black race/ethnicity, ESKD due to glomerulonephritis, hemodialysis, and predialysis nephrology care are associated with a higher likelihood of contraceptive use. The study highlights the importance of prepregnancy counseling for contraceptive use in women receiving dialysis.

## Graphical abstract

**Figure:**
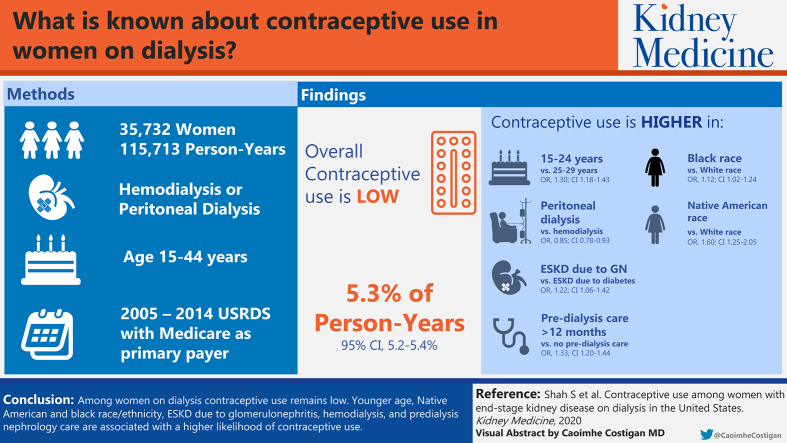

**Plain-Language Summary**

Little is known about contraceptive use in women undergoing dialysis in the United States. Using the US Renal Data System, we determined rates of contraceptive use and factors associated with contraceptive use among women undergoing dialysis. The rate of contraceptive use was low at 5.30%. Younger age, Native American and Black race/ethnicity, end-stage kidney disease due to glomerulonephritis, hemodialysis, and predialysis nephrology care were associated with a higher likelihood of contraceptive use.

Although end-stage kidney disease (ESKD) adversely affects fertility, conception is not uncommon among women receiving dialysis. ESKD increases the risk for adverse pregnancy outcomes, including preeclampsia, fetal growth restriction, and preterm delivery.^1^, ^2^, ^3^

Unplanned pregnancies occur in women with all stages of kidney disease and are associated with increased risk for obstetric complications.^4^^,^^5^ Safe and effective contraception should be made available to women who wish to delay or avoid pregnancy and those who take teratogenic medications.

Prepregnancy counseling regarding contraception for women of childbearing age with kidney disease is imperative to facilitate informed decision making.^6^ It is of paramount importance that pregnancies in this high-risk population are planned, allowing the opportunity to counsel women about the impact of pregnancy on kidney disease and the impact of kidney disease on maternal and fetal outcomes.^7^ Kidney health care providers do not routinely discuss contraception with their female patients of childbearing age.^8^^,^^9^ Little is known about the exact rates of contraceptive use and factors associated with its use among women undergoing hemodialysis and peritoneal dialysis.

We used the national ESKD registry, US Renal Data System (USRDS), to determine contraceptive use rates in women with ESKD who were receiving dialysis at any time during 2005 through 2014. The current study involves a large cohort that is not a voluntary registry and examines factors associated with contraceptive use and contraceptive rates by race/ethnicity, age, calendar year of prevalent ESKD, and dialysis modality.

## Methods

### Data Sources and Study Population

We used the USRDS database, a national registry of patients receiving maintenance dialysis, which contains information from the ESKD Medical Evidence Form of the Centers for Medicare & Medicaid Services (CMS; Form CMS-2728), as well as Medicare Part A institutional claims and Medicare Part B physician/supplier claims. USRDS is a national data system that collects information about all patients with ESKD in the United States. Data for the USRDS are compiled from the following data sources: CMS Renal Management Information System, CMS claims (incident and prevalence) data, Center for Disease Control and Prevention survey data, Standard Information Management System, Medicare evidence form (CMS-2728), ESKD death notification form (CMS-2746), and United Network for Organ Sharing transplant and wait-list data. It covers both Medicare and non-Medicare patients. CMS-2728 is the Medicare evidence form that provides demographic information for patients with ESKD.^10^

**Figure 1:**
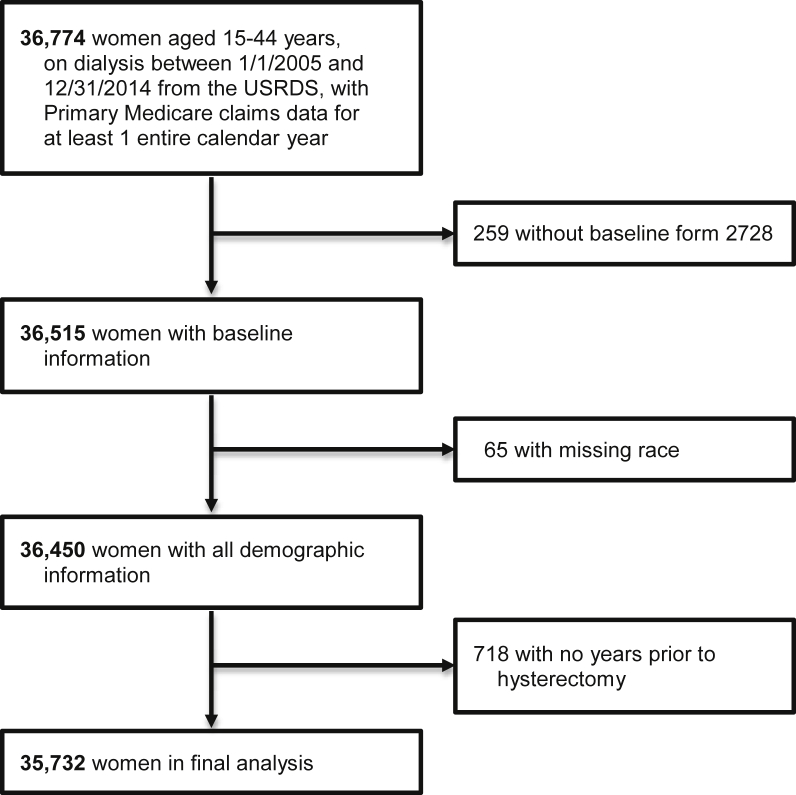
Cohort selection flow diagram. Abbreviation: USRDS, US Renal Data System.

We included women who, for at least 1 entire calendar year between 2005 and 2014, fulfilled the following criteria: were aged 15 to 44 years, were receiving dialysis, and had primary Medicare claims data. If they had multiple years that qualified, the years were entered as separate observations. The study included 115,713 person-years for 35,732 unique women. The USRDS payer history file was used to obtain information for insurance coverage for the study period. We excluded patients with no baseline CMS-2728 form in the medical evidence file. Figure 1 illustrates the study cohort derivation. Because the data were deidentified, the University of Cincinnati Institutional Review Board (IRB) committee deemed the study exempt and waived the need for informed consent (IRB approval number, 2019-0021).

We examined contraceptive use among women with ESKD receiving dialysis in the United States. Using *International Classification of Diseases, Ninth Revision, Clinical Modification* diagnosis and procedure codes and *Current Procedural Terminology, 4th Revision* codes, we searched for discharge diagnoses and medical procedures indicative of contraceptive use, which were then used to create binary indicators for each person-year for each type of contraception and for contraception overall. Prior studies have validated the specificity of this code-based method for contraception.^11^, ^12^, ^13^ Contraception was categorized into tubal ligation, intrauterine device, implant, diaphragm, injection, pill/others, and emergency contraception according to the Medicare inpatient and outpatient claims (Table S1). Women identified as having had a tubal ligation in any study year were excluded from the study for all later years. Women identified as having a hysterectomy or bilateral oophorectomy were excluded from the study for that year and all later years.

We used the patient’s file to obtain information for age, race/ethnicity, and ESKD network. Race/ethnicity was categorized as Hispanic, non-Hispanic Asian, non-Hispanic Black, non-Hispanic Native American, and non-Hispanic White. ESKD networks were classified into geographical regions of Midwest, Northeast, South, and West.^14^ The CMS-2728 form provided information on ESKD cause, body mass index (BMI), comorbid conditions (diabetes, congestive heart failure, hypertension, stroke/transient ischemic attack, and atherosclerotic heart disease), smoking history, predialysis nephrology care, and unemployment.^10^ Predialysis nephrology care was grouped into none, 12 or fewer months, more than 12 months, and unknown. The treatment history file was used to obtain information on dialysis modality and duration of dialysis, measured as number of years receiving dialysis before a given date. Dialysis modalities were classified into hemodialysis and peritoneal dialysis. Information for zip code of residence was obtained from the residence file and combined with zip code–level data from the US Census Bureau American Community Survey 5-year estimates (2007-2011) to determine the neighborhood poverty level, defined by percent of zip code residents living below the federal poverty level, and was grouped into 5 categories: I (<13.8%), II (13.8%-19.9%), III (20.0%-39.9%), IV (≥40%), and unknown.^15^^,^^16^ For continuous measures of age, BMI, and time receiving dialysis, we created groups based on clinical relevance. Multiple imputation was used to account for unavailable information for covariates.

### Statistical Analysis

Summary statistics are presented as percentages for categorical data and mean ± standard deviation for continuous variables. We tested differences between groups using χ^2^ tests for categorical variables and *t* tests for continuous variables. Statistical significance was set at 2-tailed *P* = 0.05, unadjusted for multiple tests. We determined unadjusted rates of contraceptive use, expressed as percent of person-years in which at least 1 indicator of contraceptive use appeared, for the overall cohort and by race/ethnicity, age, calendar year of prevalent ESKD, and type of dialysis modality.

Contraception codes determined the presence of contraception use but not its timing. Therefore, we used multivariable logistic regression for individual years of prevalent ESKD after dialysis initiation to determine factors associated with contraceptive use. Subject was treated as a repeated effect, and a compound symmetric correlation matrix was used. Multivariable logistic models were nonparsimonious and included the covariates of age, race/ethnicity, BMI, calendar year of prevalent ESKD, ESKD cause, neighborhood poverty level, geographical region, dialysis modality, time receiving dialysis, predialysis nephrology care, comorbid conditions, history of smoking, and employment status. Age, calendar year of prevalent ESKD, neighborhood poverty level, dialysis modality, and time receiving dialysis were determined from the beginning of each calendar year. Risk estimates for contraceptive use were expressed as odds ratios (ORs) and their 95% CIs. Data were analyzed using SAS, version 9.4 (SAS Institute).

## Results

### Baseline Demographics and Clinical Characteristics

The study cohort included 115,713 observed person-years for 35,732 women. Among women receiving dialysis, the rate of any contraception use during the study period was 5.30% (95% CI, 5.17%-5.42%) person-years, during which at least 1 contraception was used. Table 1 shows characteristics of the women in the cohort, separated by those who did and did not use contraception at all during the study period. Mean ages at study entry were 34 ± 7 years for women without contraceptive use and 30 ± 7 years for women with any contraceptive use. Compared with women with no contraceptive use, women with any contraceptive use had a higher proportion of Native Americans (1.7% vs 2.4%) and a lower proportion of Whites (28.6% vs 26.0%). Glomerulonephritis was the most common cause of ESKD (25.0%) among those with contraceptive use, and diabetes was the most common cause of ESKD among women without contraceptive use (28.7%). Compared with women without contraceptive use, women with any contraceptive use had lower percentages of comorbid conditions of diabetes mellitus (29.3% vs 21.6%), congestive heart failure (10.8% vs 8.8%), cerebrovascular accident/transient ischemic attack (2.7% vs 1.6%), atherosclerotic heart disease (2.1% vs 1.2%), and smoking (6.2% vs 4.5%).

**Table 1:** Baseline Characteristics of Women With ESKD on Dialysis Separated by Contraceptive Use During the Follow-up Period

Characteristics	Without Contraception (N = 31,632)	With Contraception (N = 4,100)	*P*^a^
Demographics			
Age, y^b^^,^^c^	34 ± 7	30 ± 7	<0.001; <0.001
15-24	3,046 (9.6%)	1,020 (24.9%)	
25-29	3,917 (12.4%)	977 (23.8%)	
30-34	6,157 (19.5%)	904 (22.0%)	
35-39	8,780 (27.7%)	798 (19.5%)	
40-44	9,732 (30.8%)	401 (9.8%)	
Race/ethnicity			<0.001
Asian	1,332 (4.2%)	169 (4.1%)	
Black	15,180 (48.0%)	2,017 (49.2%)	
Hispanic	5,545 (17.5%)	749 (18.3%)	
Native American	524 (1.7%)	97 (2.4%)	
White	9,051 (28.6%)	1,068 (26.0%)	
BMI, kg/m^2^ (84.8% nonmissing)^b^	30.0 ± 9.4	29.5 ± 9.4	0.003; <0.001
<18.5	1,364 (4.3%)	238 (5.8%)	
18.5-25	8,241 (26.1%)	1,109 (27.0%)	
25.1-30	6,080 (19.2%)	777 (19.0%)	
>30	11,986 (37.9%)	1,531 (37.3%)	
Missing	3,961 (12.5%)	445 (10.9%)	
Cause of ESKD			<0.001
Cystic/hereditary	1,610 (5.1%)	243 (5.9%)	
Diabetes mellitus	9,071 (28.7%)	826 (20.2%)	
GN	5,847 (18.5%)	1,026 (25.0%)	
Hypertension/LVD	6,467 (20.4%)	693 (16.9%)	
Interstitial nephritis/pyelonephritis	1,063 (3.4%)	159 (3.9%)	
Malignancy	1,044 (3.3%)	172 (4.2%)	
Secondary GN/vasculitis	3,994 (12.6%)	600 (14.6%)	
Others	2,536 (8.0%)	381 (9.3%)	
Neighborhood poverty level^c^			0.26
<13.8%	14,941 (47.2%)	1,866 (45.6%)	
13.8%-20%	6,599 (20.9%)	881 (21.5%)	
>20%-40%	8,726 (27.6%)	1,182 (28.8%)	
>40%	720 (2.3%)	92 (2.2%)	
Missing	646 (2.0%)	79 (1.9%)	
Geographic region			<0.001
Midwestern	6,103 (19.3%)	908 (22.1%)	
Northeastern	4,686 (14.8%)	519 (12.7%)	
Southern	15,530 (49.1%)	1,903 (46.4%)	
Western	5,304 (16.8%)	769 (18.8%)	
Missing	9 (0.0%)	0 (0.0%)	
Dialysis modality^c^			0.36
Peritoneal dialysis	5,762 (18.2%)	723 (17.6%)	
Hemodialysis	25,870 (81.8%)	3,377 (82.4%)	
Time on dialysis^c^			<0.001
<2 y	19,628 (62.0%)	2,724 (66.4%)	
2-<5 y	7,777 (24.6%)	909 (22.2%)	
5-<10 y	3,090 (9.8%)	359 (8.8%)	
≥10 y	1,137 (3.6%)	108 (2.6%)	
Predialysis nephrology care			<0.001
None	5,719 (18.1%)	677 (16.5%)	
≤12 mo	6,165 (19.5%)	845 (20.6%)	
>12 mo	5,312 (16.8%)	798 (19.5%)	
Missing	14,436 (45.6%)	1,780 (43.4%)	
Comorbid conditions			
Diabetes mellitus	9,260 (29.3%)	885 (21.6%)	<0.001
Congestive heart failure	3,426 (10.8%)	360 (8.8%)	<0.001
Hypertension/LVD	24,946 (78.9%)	3,180 (77.6%)	0.06
Smoking	1,969 (6.2%)	185 (4.5%)	<0.001
TIA/cerebrovascular accident	869 (2.7%)	66 (1.6%)	<0.001
Atherosclerotic heart disease	671 (2.1%)	51 (1.2%)	<0.001
Employment status			
Unemployed	13,918 (44.0%)	1,874 (45.7%)	0.04

Abbreviations: BMI, body mass index; ESKD, end-stage kidney disease; GN, glomerulonephritis; LVD, large-vessel disease; TIA, transient ischemic attack.

a For age and BMI, the first *P* value compares the data as continuous values and the second *P* value examines comparisons across groups.

b Reported in mean ± standard deviation; all others are reported as percentages of person-years. Women with contraception refers to women who ever used contraception during the study period (11.5%), and women without contraception refers to women who never used contraception during the study period.

c Values associated with women’s first eligible year for study.

### Contraceptive Rates in Women With ESKD Receiving Dialysis

**Figure 2:**
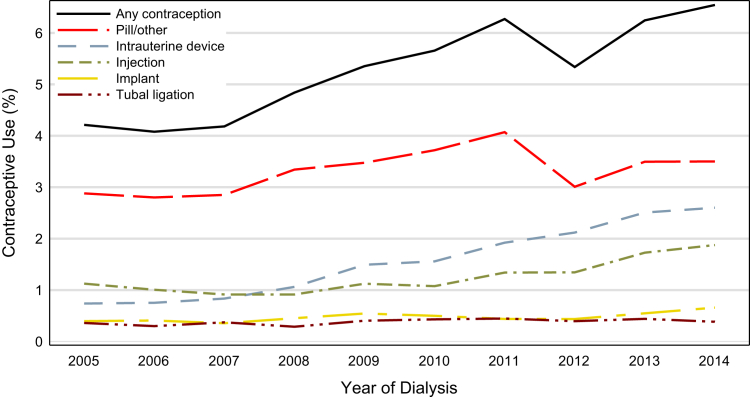
Rates of types of contraceptives use among women with end-stage kidney disease receiving dialysis from 2005 to 2014. Any contraception shows a fairly steady increase across the study time; all categories increase or remain constant from beginning to end. Emergency contraception and diaphragm had rates of <0.10% for all years, not shown in the graph.

The rate of any form of contraceptive use in women with ESKD during the study period was 5.30% (95% CI, 5.17%-5.42%). Overall, rates of different types of contraceptive use were as follows: intrauterine device insertions (1.58%; 95% CI, 1.51%-1.65%), injection (1.25%; 95% CI, 1.19%-1.32%), implant (0.48%; 95% CI, 0.44%-0.51%), tubal ligation (0.38%; 95% CI, 0.35%-0.42%), diaphragm (0.01%; 95% CI, <0.01%-0.1%), emergency contraception (0.02%; 95% CI, 0.01%-0.02%), and pills/others (3.32%; 95% CI, 3.22%-3.43%). Contraceptive use increased from 2005 to 2014 (4.21%; 95% CI, 3.84%-4.59% vs 6.54%; 95% CI, 6.10%-6.99%). From 2005 to 2014, the rate of intrauterine device insertions increased from 0.74% (95% CI, 0.58%-0.90%) to 2.60% (95% CI, 2.32%-2.89%), implant insertion increased from 0.40% (95% CI, 0.28%-0.51%) to 0.66% (95% CI, 0.51%-0.80%), rate of tubal ligation stayed fairly constant (0.36%; 95% CI, 0.25%-0.47% vs 0.38%; 95% CI, 0.27%-0.49%), and pills/others increased from 2.88% (95% CI, 2.57%-3.19%) to 3.50% (95% CI, 3.17%-3.83%; Fig 2).

**Figure 3:**
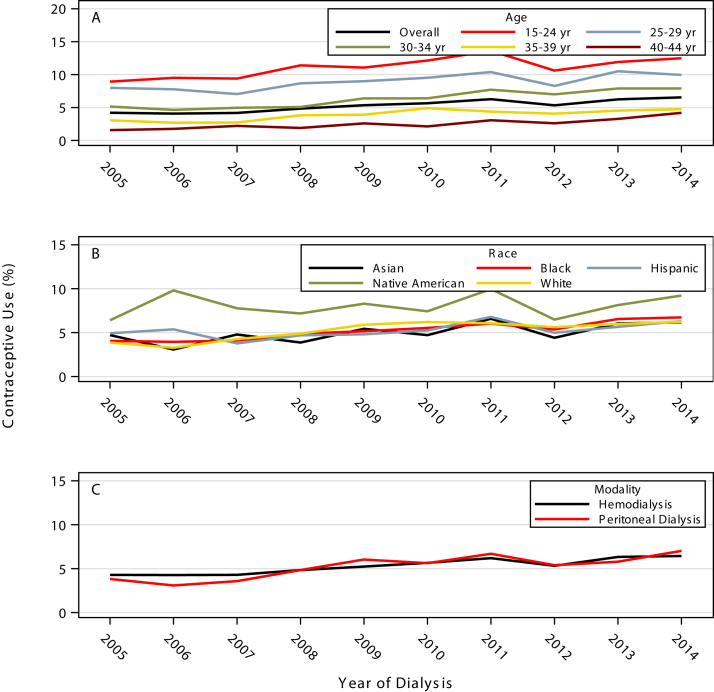
Contraceptive rates in women with end-stage kidney disease by: (A) age, (B) race, and (C) dialysis modality. All 3 graphs show an upward trend from 2005 to 2014. The age graph shows a decline in rates with increasing age. The race graph shows a higher rate among Native Americans as compared with other races. The dialysis modality graph shows similar rates across time from 2005 to 2014.

Among 5 racial/ethnic groups, Native American women had the highest rate of contraceptive use (8.08%; 95% CI, 6.91%-9.26%), followed by Hispanic women (5.29%; 95% CI, 4.98%-5.60%), Black women (5.25%; 95% CI, 5.07%-5.43%), White women (5.23%; 95% CI, 4.98%-5.48%), and Asian women (5.05%; 95% CI, 4.43%-5.66%). All races had an increase in contraceptive use from 2005 to 2014. From 2005 to 2014, the rate of contraceptive use increased among Asian women from 4.72% (95% CI, 2.70%-6.73%) to 6.17% (95% CI, 4.19%-8.15%), among Black women from 4.06% (95% CI, 3.54%-4.57%) to 6.73% (95% CI, 6.10%-7.37%), among Hispanic women from 4.92% (95% CI, 3.92%-5.92%) to 6.30% (95% CI, 5.30%-7.31%), among Native American women from 6.40% (95% CI, 3.04%-9.77%) to 9.22% (95% CI, 5.37%-13.07%), and among White women from 3.86% (95% CI, 3.18%-4.55%) to 6.23% (95% CI, 5.37%-7.09%; Fig 3A).

With regard to age, the rate of contraceptive use increased from 2005 to 2014 across all age groups. Contraceptive use was highest among women aged 15 to 24 years (11.11%; 95% CI, 10.50%-11.72%) and lowest among women aged 40 to 44 years (2.56%; 95% CI, 2.40%-2.73%; Fig 3B). Figure 3C shows rates of contraceptive use by type of dialysis modality. Among women receiving hemodialysis, contraceptive use increased from 4.29% (95% CI, 3.88%-4.70%) in 2005 to 6.44% (95% CI, 5.96%-6.93%) in 2014. Among women receiving peritoneal dialysis, contraceptive use increased from 3.82% (95% CI, 2.94%-4.70%) in 2005 to 7.03% (95% CI, 5.92%-8.13%) in 2014.

### Factors Associated With Contraceptive Use in Women With ESKD Receiving Dialysis

Table 2 elucidates factors associated with the likelihood of contraceptive use among women with ESKD from the adjusted logistic regression model. Compared with women aged 25 to 29 years, contraceptive use was higher in women aged 15 to 24 years (OR, 1.30; 95% CI, 1.18-1.43) and lower in women aged 30 to 34 years (OR, 0.74; 95% CI, 0.68-0.81), 35 to 39 years (OR, 0.46; 95% CI, 0.42-0.50), and 40 to 44 years (OR, 0.30; 95% CI, 0.27-0.34). As compared with White women, contraceptive use was higher in Black (OR, 1.12; 95%, 1.02-1.24) and Native American women (OR, 1.60; 95% CI, 1.25-2.05). There was an upward linear trend associated with calendar year of prevalent ESKD (OR, 1.07 for 1-year increase; 95% CI, 1.06-1.08). Women with ESKD due to glomerulonephritis had a higher likelihood of contraceptive use (OR, 1.22; 95% CI, 1.06-1.42) than women with ESKD due to diabetes. Compared with women residing in the Southern geographical region, contraceptive use was higher in women residing in the Midwestern (OR, 1.24; 95% CI, 1.13-1.36) and Western regions (OR, 1.18; 95% CI, 1.06-1.31). Women receiving peritoneal dialysis had a lower likelihood of contraceptive use than women receiving hemodialysis (OR, 0.85; 95% CI, 0.78-0.93). Compared with women with no predialysis nephrology care, contraceptive use was higher in women who received nephrology care for 12 or fewer months (OR, 1.22; 95% CI, 1.09-1.37) and more than 12 months (OR, 1.33; 95% CI, 1.20-1.47). The comorbid condition of hypertension/large-vessel disease was associated with a higher likelihood of contraceptive use (OR, 1.14; 95% CI, 1.04-1.24), whereas the comorbid condition of cerebrovascular accident/transient ischemic attack was associated with a lower likelihood of contraceptive use (OR, 0.71; 95% CI, 0.55-0.93). History of smoking was associated with a lower likelihood of contraceptive use (OR, 0.80; 95% CI, 0.67-0.94). BMI, neighborhood poverty level, diabetes mellitus, and unemployment were not significantly associated with contraceptive use among women with ESKD.

**Table 2:** Factors Associated With Contraceptive Use in Women With ESKD on Dialysis

Variable	Odds Ratio (95% CI)	*P*
Age, y		<0.001
15-24	1.30 (1.18-1.43)	
25-29	Reference	
30-34	0.74 (0.68-0.81)	
35-39	0.46 (0.42-0.50)	
40-44	0.30 (0.27-0.34)	
Race/ethnicity		0.001
White	Reference	
Asian	0.92 (0.77-1.11)	
Black	1.12 (1.02-1.24)	
Hispanic	1.01 (0.90-1.14)	
Native American	1.60 (1.25-2.05)	
BMI, kg/m^2^		0.82
18.5-25	Reference	
<18.5	1.02 (0.88-1.18)	
25.1-30	1.03 (0.93-1.14)	
>30	1.05 (0.97-1.14)	
Calendar year of prevalent ESKD	1.07 (1.06-1.08)	<0.001
Cause of ESKD		<0.001
Diabetes mellitus	Reference	
Cystic/hereditary	1.07 (0.88-1.30)	
GN	1.22 (1.06-1.42)	
Hypertension/LVD	0.96 (0.83-1.11)	
Interstitial nephritis/pyelonephritis	1.13 (0.90-1.42)	
Malignancy	1.23 (1.00-1.53)	
Secondary GN/vasculitis	1.04 (0.89-1.21)	
Others	1.24 (1.05-1.47)	
Neighborhood poverty		0.61
<13.8%	Reference	
13.8%-20%	1.04 (0.96-1.13)	
>20%-40%	0.96 (0.89-1.05)	
>40%	0.97 (0.77-1.22)	
Geographic region		<0.001
Southern	Reference	
Midwestern	1.24 (1.13-1.36)	
Northeastern	0.93 (0.83-1.04)	
Western	1.18 (1.06-1.31)	
Dialysis modality		<0.001
Hemodialysis	Reference	
Peritoneal dialysis	0.85 (0.78-0.93)	
Time on dialysis		0.39
<2 y	Reference	
2-<5 y	0.96 (0.90-1.02)	
5-<10 y	0.90 (0.83-0.98)	
≥10 y	0.81 (0.70-0.93)	
Predialysis nephrology care		<0.001
None	Reference	
≤12 mo	1.22 (1.09-1.37)	
>12 mo	1.33 (1.20-1.47)	
History of transplant	0.95 (0.86-1.06)	0.92
Diabetes mellitus	0.89 (0.79-1.01)	0.08
Congestive heart failure	0.96 (0.85-1.08)	0.42
Hypertension/LVD	1.14 (1.04-1.24)	0.01
Smoking	0.80 (0.67-0.94)	0.004
TIA/cerebrovascular accident	0.71 (0.55-0.93)	0.01
Atherosclerotic heart disease	0.92 (0.68-1.26)	0.60
Unemployed	1.05 (0.97-1.13)	0.26

Abbreviations: BMI, body mass index; ESKD, end-stage kidney disease; GN, glomerulonephritis; LVD, large-vessel disease; TIA, transient ischemic attack.

## Discussion

Using a national registry of patients with ESKD, we found that although contraceptive use rates increased in the last decade, only 5% of women of childbearing age used any form of contraception during a year receiving dialysis. To our knowledge, this is one of the first reports in the United States to elucidate rates and racial differences in contraceptive use among women receiving dialysis. Age, cause of ESKD, dialysis modality, predialysis nephrology care, geographical region, and smoking history are other important factors associated with a higher likelihood of contraceptive use in our study.

The present study reports an overall contraceptive use rate of 5.30% in women receiving dialysis in the United States during the study period. Although no published contemporary data exist for contraceptive use among dialysis patients, in the general population, 64.9% of women aged 15 to 49 years in the United States used a method of contraception between 2015 and 2017.^17^ The lower rates in the dialysis population may be attributed to the overrepresentation of older women in the cohort and lack of counseling for need of contraception for women of childbearing age who are sexually active and not planning pregnancies. In a questionnaire completed by 76 women who were 55 years or younger at the start of dialysis, results showed that despite 50% of women being sexually active, 36% used contraception and only 13% had had a discussion with their nephrologist about possible pregnancy and contraception.^1^ A recent survey by Sachdeva et al^8^ showed that only 46% of kidney care providers counsel their female dialysis patients about contraception. Therefore, contraceptive counseling and provision of safe and effective contraception remain inadequate for many women with ESKD.

The current study shows significant racial/ethnic differences in contraceptive use among women receiving dialysis. Compared with White women undergoing dialysis, the likelihood of contraceptive use was higher by 60% and 12% in Native American and Black women, respectively. The findings persisted even after adjustment for socioeconomic status and neighborhood poverty. Differences in cultural beliefs, social values, family support systems, and health literacy between different races/ethnicity may have contributed to the observed differences in contraceptive use in women undergoing dialysis in the present study. Health literacy and education empower women with autonomy in making fertility-related decisions and have shown to be associated with contraceptive use.^18^ Education level is not captured as a patient-level variable in the USRDS and remains a limitation of our study.

In our study, women receiving peritoneal dialysis had a 15% lower likelihood of contraceptive use than women receiving hemodialysis. Peritoneal dialysis reduces a woman’s chances of getting pregnant by 50% compared with hemodialysis.^19^^,^^20^ Lower conception may be related to the use of hypertonic dextrose solution fluid in the peritoneal cavity, which interferes with the transit of ova to the fallopian tube.^21^ The lower rates of conception in women receiving peritoneal dialysis may lead to the lower contraceptive use among women receiving peritoneal dialysis. However, pregnancy is not uncommon among women receiving peritoneal dialysis, and contraception should be discussed with all women of reproductive potential and contraceptive use should be advised until active pregnancy is planned regardless of dialysis modality status.

Another finding of our study was that predialysis nephrology care was associated with a 20% to 40% higher likelihood of contraceptive use than in women with no predialysis care. Predialysis nephrology care is associated with better outcomes among patients with ESKD. For example, seeing a nephrologist before initiation of dialysis leads to higher use of permanent vascular access at dialysis initiation.^22^ Nephrologists during clinic visits do not routinely discuss conception with patients with chronic kidney disease.^23^ Currently, there are no specific recommendations or guidelines for the use of contraceptives in women undergoing dialysis. There is a need for concerted efforts to guide kidney health care providers in the reproductive health management for women with kidney disease. The present study underscores the importance of reproductive health counseling among patients with ESKD, especially in the predialysis period that may lead to higher contraceptive use and possibly prevent unintentional pregnancies.

In our study, intrauterine device and pill/others were the most common methods of contraception for use in women receiving dialysis. In the general population, the most common contraceptive methods include female sterilization (18.6%), oral contraceptive pill (12.6%), and long-acting reversible contraceptives (10.3%).^17^ The decision to proceed with or delay conception is highly individualized and is affected by the baseline health of the woman, the woman’s age and comorbid conditions, and the anticipated wait time for a donor kidney, if applicable. Contraceptive decision making therefore needs to balance patient acceptability and safety. Estrogen-containing methods, including oral pills, the transdermal patch, and vaginal rings, are less preferred in patients with ESKD because of the markedly increased cardiovascular risk in patients with ESKD and their association with increased blood pressure and thrombotic and vascular events. For women receiving dialysis, progesterone-only methods have a better safety profile, including the progesterone-only pill (desogestrel preparations), the levonorgestrel-releasing intrauterine devices, and the etonogestrel implant.^24^

A significant strength of our study is that it includes a large number of women of childbearing age with ESKD receiving dialysis in the United States, thus providing us with information on contraceptive use in a heterogeneous population. Additionally, we have identified particular factors that may be considered while counseling women with ESKD. This will help in making future guidelines for follow-up and management of reproductive health among women receiving dialysis.

Limitations of our study include the observational design, which makes it difficult to identify possible interventions that could affect contraceptive practices, and the use of deidentified data. Patient-level variables of education, which may affect contraceptive use, are not captured on Form 2728 and were not available for our analysis. ESKD is associated with menstrual irregularities such as premature menopause, but we were not able to account for premature menopause in the present study due to lack of administrative diagnostic claims. Neither could we account for procedures that affect fertility, such as hysterectomies or tubal ligations before 2005. The study also could not account for exact rates of oral contraceptive use due to lack of pharmacy claims data or the impact of methods that last for multiple years, such as intrauterine device or injection. We were not able to determine sexual activity levels in women and contraceptive methods used in men. However, importantly, it accounts for all contraceptive use in women and not just that reported in the voluntary registries, thereby predicting accurate contraceptive rates among women with ESKD.

In conclusion, our study demonstrates a significant increase in rates of contraceptive use in dialysis patients in the recent decade in the United States, significant differences across race, and a significant effect of predialysis nephrology care on the likelihood of contraceptive use. Pregnancy in a patient with ESKD is associated with higher risk for maternal and fetal complications, highlighting the importance of pregnancy planning and contraceptive counseling in women receiving dialysis. Finally, the present study emphasizes the importance of formulating policies that promote awareness of reproductive health and contraception among women with ESKD receiving dialysis.
