# Supplementary material for: Contraceptive Use Among Women With End-Stage Kidney Disease on Dialysis in the United States
Source: Kidney Med. 2020 Oct 27;2(6):707–715.e1. doi: 10.1016/j.xkme.2020.08.010 (PMC7729239; doi:10.1016/j.xkme.2020.08.010)
Supplement: Supplementary File (PDF) — Table S1. [file mmc1.pdf]

Table S1. Discharge diagnoses and medical procedures indicative of contraceptive use or exclusions

Type of Contraception	CPT:	ICD diagnoses:	ICD procedures:
Tubal Ligation		6282T 9989T V252T V5042	6621 6622 6629 6631 6632 6639 6640 6651 6652 6661 6662 6663 6692 6697
Intrauterine Device	58300 58301 J7300 J7301 J7302	99632 V251 V2511 V2512 V2513 V253 V2542 V259 V4551	697
Implant	11975 J7307	99676 V2543 V255 V2551 V4552	
Diaphragm	57170 A4261 A4266 A4267 A4268 A4269		
Injection	J1055		
Pill/Other	J7303 J7304 S4993	V250 V2500 V2501 V2502 V2504 V2509 V2521 V2540 V2541 V2549 V258	
Emergency		V2503	
Exclusions			
Hysterectomy	58150 58151 58152 58153 58154 58185 58156 58157 58158 58159 58180 58200 58210 58240 58260 58261 58262 58263 58264 58265 58266 58267 58268 58269 58270 58271 58272 58273 58274 58275 58276 58277 58278 58279 58280 58281 58282 58283 58284 58285 58286 58287 58288 58289 58290 58291 58292 58293 58294 58295 58296 58297 58298 58299 58540 58541 58542 58543 58544 58545 58546 58547 58548 58549 58550 58551 58552 58553 58554 58555 58556 58557 58558 58559 58570 58571 58572 58573 58574 58575 58576 58577 58578 58579	V8801	683 6831 684 6851 6859 687 688 689
Oophorectomy			6551 6552 6553 6554 6561 6562 6563 6564
